# 

**DOI:** 10.29045/14784726.2018.09.3.2.29

**Published:** 2018-09-01

**Authors:** Matt Holland

**Affiliations:** Librarian, Library and Knowledge Service for NHS Ambulance Services in England

Dear Editors,

A new library service for ambulance services in England has been set up. This service is a partnership between seven ambulance services (East Midlands, West Midlands, North East, North West, Yorkshire, East of England and South Central) to provide a basic library service for staff.

The library is a virtual service operating from the website URL: http://ambulance.libguides.com and is managed by the librarian, Matt Holland. This service builds on the successful model that was previously operated at NWAS.

The library provides four core services:

- **Request an article:** complete an online form and request any article you need.
- **Request a search:** complete the online form to request a literature search.
- **Current awareness:** sign up for monthly current awareness updates.
- **Guides and help:** the library has written guides on topics related to searching, research and paramedic practice.

The library aims to provide information on a range of topics relevant to ambulance services, and is not restricted to purely clinical and management subject areas. We are working with Manchester University NFT Trust Library Services to provide a first-class document supply service to underpin the *Request an article* service and we are confident we will be able to supply any requests for journal articles.

